# Human infections with Shiga toxin-producing Escherichia coli other than serogroup O157 in Germany.

**DOI:** 10.3201/eid0404.980415

**Published:** 1998

**Authors:** L Beutin, S Zimmermann, K Gleier

**Affiliations:** Robert Koch-Institut, Abt. Mikrobiologie, Berlin, Germany. BeutinL@rki.de

## Abstract

We investigated different types of Shiga toxin-producing Escherichia coli (STEC) not belonging to serogroup O157 for their role as human pathogens. Non-O157 STEC isolated from 89 human patients in Germany were characterized according to serotypes, virulence markers, and association with human illness. EaeA-positive STEC were isolated from 54 (60.7%) of the patients and were frequently associated with severe diarrheal disease, hemolytic uremic syndrome, and young age. EaeA-negative STEC were found in 35 (39.3%) of the patients and were more associated with clinically uncomplicated cases and adult patients. For pediatric patients, a serotype-independent diagnosis of STEC is recommended.


### Human Infections with Shiga Toxin-Producing *Escherichia coli* Other Than Serogroup O157 in Germany

**Lothar Beutin, Sonja Zimmermann, and Kerstin Gleier**
Robert Koch-Institut, Berlin, Germany

We investigated different types of Shiga toxin-producing *Escherichia coli* (STEC) not belonging to serogroup O157 for their role as human pathogens. Non-O157 STEC isolated from 89 human patients in Germany were characterized according to serotypes, virulence markers, and association with human illness. *Eae*A-positive STEC were isolated from 54 (60.7%) of the patients and were frequently associated with severe diarrheal disease, hemolytic uremic syndrome, and young age. *Eae*A-negative STEC were found in 35 (39.3%) of the patients and were more associated with clinically uncomplicated cases and adult patients. For pediatric patients, a serotype-independent diagnosis of STEC is recommended.

Certain strains of *Escherichia coli* belonging to different O-(LPS) and H-(flagellar) serotypes produce potent cytotoxins called Shiga toxins (Stx) (1). The natural hosts of Shiga toxin-producing *E. coli* (STEC) are farm and wildlife ruminants. In humans, STEC can cause disease, although the clinical picture may vary from uncomplicated diarrhea, to hemorrhagic colitis (HC), to hemolytic uremic syndrome (HUS) (2,3). Large outbreaks and cases of HC and HUS in humans were mainly associated with STEC strains belonging to serogroup O157. Human infections with STEC O157 are under nation-wide surveillance in a number of countries, but the detection of non-O157 STEC infections is often limited to a small number of specialized laboratories because STEC O157 colonies are more easily detectable on some culture media than non-O157 STEC types, which are thus often missed in laboratory diagnosis of stool specimens (4,5). However, humans are likely more exposed to non-O157 STEC because these strains are more prevalent in animals and as contaminants in foods than STEC O157 (4,6). Infections with some non-O157 STEC types, such as O26 and O111, are associated with illness in humans, but the role of other non-O157 STEC types in human disease needs further examination (3,7). In Germany, the number of clinical laboratories performing diagnosis of STEC from stool samples has increased since immunologic assays (Stx-enzyme-linked immunosorbent assay [ELISA]) specific for detection of Stx1 and Stx2 became commercially available. The Stx-specific ELISA kits are useful for detection of different serotypes of STEC; consequently, more non-O157 STEC-positive stool specimens and bacterial isolates were sent to our laboratory for confirmation and typing. In this study, we report on non-O157 STEC infections in 89 human patients infected during 1996 in Germany. We investigated the relationship between STEC serotype, STEC virulence factors (such as the *eae*A gene, production of enterohemolysin), Stx1 and Stx2, and the clinical signs in the patients.

Address for correspondence: Lothar Beutin, Robert Koch-Institut, Abt. Mikrobiologie, Nordufer 20, D-13353 Berlin, Germany; fax: 47-304547-2673; e-mail: BeutinL@rki.de.

### Isolation of Non-O157 STEC Strains

Of 89 non-O157 STEC isolates from 89 patients, 46 were isolated in the collaborating laboratories and 43 in our laboratory from stool samples. STEC isolates or stool specimens were sent together with patient data (age, gender, and illness) from 37 private, hospital, and public health laboratories from rural and urban areas of Germany. In our laboratory, a small sample of stool was injected into 5 ml of Tryptic Soy Broth and incubated overnight at 37°C (8). Aliquots from the grown stool culture were tested for toxicity and for Stx-specific DNA sequences by polymerase-chain reaction (PCR) (9,10). STEC were isolated from Stx-positive stool cultures by combined use of enterohemolysin agar and the STEC-RPLA test (8). Enterohemolysin-negative STEC were identified by testing different coliform colonies with the STEC-RPLA assay. All stool cultures were additionally plated on Sorbitol MacConkey Agar (SMAC) for identification of sorbitol-nonfermenting STEC O157 strains.

Most of our collaborating laboratories in Germany used Stx-specific ELISA for routine examination of stool samples. Examination for STEC was performed in cases of diarrhea, HUS, or on special request; diagnostic approaches differed among laboratories. Some supplied only Stx-ELISA-positive stool specimens for further examination; others attempted to isolate STEC colonies by using combinations of commercially available diagnostic tools such as Stx-ELISA, STEC-RPLA, agglutinating rabbit antisera for enteropathogenic *E. coli* (EPEC) O-groups including serogroup O157, enterohemolysin agar, and SMAC. Of the 37 collaborating laboratories, two used their own methods, such as the Stx-toxicity test or an Stx-specific PCR for identification of STEC from stool.

In our laboratory, all 89 non-O157 STEC isolates were tested for cytotoxicity by the Stx test and for production of Stx1 and Stx2 by the STEC-RPLA assay. The enterohemolytic phenotype was determined on enterohemolysin agar (8). The presence of the *eae*A gene was determined by PCR using two different primer pairs specific for the conserved region of the *eae*A gene (11,12). Serotyping of O- and H-antigens of *E. coli* strains was performed as described (13).

### Characterization of Non-O157 STEC Strains

Only one STEC serotype was isolated from each of the 89 patients except for one patient with HC who excreted simultaneously STEC types O157:H7 and O26:H11. Of the 89 STEC strains, 69 (77.5%) could be typed to 15 different O-groups, and 20 were O-untypable or O-rough (Table 1). The STEC strains varied in H-antigens, which were conserved within some of the O-groups, and 31 STEC strains (34.8%) were nonmotile. Most isolates (n = 59) produced only Stx1 (66.3%) and 11 (12.4%) only Stx2. Production of Stx1 and Stx2 was found in 19 (21.3%) strains and was associated with strains of O-groups 113 and 145. Fifty-four (60.7%) of the STEC strains were found *eae*A-positive. The *eae*A-gene was present in all STEC strains belonging to some common serogroups (O26, O103, O111, O118, and O145) and in 8 of 17 STEC O-rough strains. The enterohemolytic phenotype was expressed in 82 strains (92.1%) and was highly associated with *eae*A positive STEC (53 [98.2%] of 54 were positive). All STEC strains were positive for fermentation of sorbitol.

### Relationship Between STEC Types, Virulence Markers, and Clinical Status of the Patients

Fifty-one (60.7%) of the patients were female, and 33 (39.3%) were male; the gender of five patients was not reported. Of 85 patients whose ages were known, 46 (54.1%) were 3 months to 6 years of age, 10 (11.8%) were 6 to 15 years of age, and 29 (34.1%) were older than 15 years (Table 2). Patients were divided into four groups according to case history (Table 1). Six cases (6.7%) of asymptomatic excretors of STEC were detected by routine screening of stool specimens. Nonbloody diarrhea, in some cases accompanied with abdominal pain and vomiting, was reported for 72 (80.9%) patients. Bloody diarrhea was reported in 7 (7.9%) and HUS in 4 (4.5%) cases. Episodes of protracted, nonbloody diarrhea for 2 weeks or more were reported for 11 (12.4%) patients. Double infections with STEC and other diarrheal pathogens were not reported except for 6 (6.7 %) patients who were additionally infected with *Salmonella enterica* sp. (Table 1).

The clinical disease was not associated with the Stx types produced by the infecting strains. In contrast, the *eae*A-gene was closely associated with severe illness and young age (Tables 1, 2). Thus, 9 (81.8%) of the 11 patients with bloody diarrhea or HUS were infected with *eae*A-positive STEC of serogroups O26, O103, O111, O118, or O145; 8 (72.7%) of 11 patients with protracted, nonbloody diarrhea carried *eae*A-positive STEC of serogroups O118, O145, O163, and O-rough. Of 54 patients with *eae*A-positive STEC isolates, 41 (75.9%) were younger than 6 years of age with a peak age of 1 to 3 years (Table 2) and a mean age of 8 years and 9 months. In contrast, *eae*A-negative STEC were more prevalent in adults and were isolated from 35 patients; age was known in 31 (Table 2). Only five (16.1 %) were younger than 6 years of age, and the mean age in this group was 27 years and 10 months.

Most of the non-O157 STEC infections were sporadic, and the sources of infection

Table 1. Non-O157 STEC infections in human patients in Germany, 1996

| STEC serotypes | | No. of STEC strains with properties | | | | | Clinical status | | | |
|---|---|---|---|---|---|---|---|---|---|---|
| O-group[a] (no. of strains) | H-types[b] | Stx1 | Stx2 | Stx1+ Stx2 | *eae*A | Entero-hemo-lytic | Asympto-matic | Non-bloody diarrhea | Bloody diarrhea | HUS[c] |
| O5 (1) | NM | 0 | 0 | 1 | 0 | 1 | 0 | 1 | 0 | 0 |
| O18 (1) | H15 | 0 | 0 | 1 | 1 | 1 | 0 | 1[d] | 0 | 0 |
| O26 (20) | H11, NT, NM | 16 | 4 | 0 | 20 | 19 | 1 | 17 | 2[d,e] | 0 |
| O76 (3) | H19 | 3 | 0 | 0 | 0 | 3 | 0 | 3 | 0 | 0 |
| O78 (1) | NM | 0 | 1 | 0 | 0 | 1 | 1 | 0 | 0 | 0 |
| O84 (1) | NT | 1 | 0 | 0 | 1 | 1 | 0 | 1[d] | 0 | 0 |
| O91 (4) | H14, NM | 3 | 0 | 1 | 0 | 4 | 0 | 4 | 0 | 0 |
| O103 (7) | H2, H7, NT | 6 | 1 | 0 | 7 | 7 | 0 | 5[d] | 1 | 1 |
| O111 (3) | NT | 1 | 1 | 1 | 3 | 3 | 1 | 1 | 0 | 1 |
| O113 (3) | H32, NT | 0 | 0 | 3 | 0 | 3 | 0 | 3 | 0 | 0 |
| O118 (8) | H12, H16 NT | 7 | 1 | 0 | 7 | 7 | 0 | 6[d] | 1 | 1 |
| O128 (5) | H2, NM | 3 | 0 | 2 | 0 | 5 | 1 | 4 | 0 | 0 |
| O145 (6) | H28, NM | 1 | 0 | 5 | 6 | 6 | 0 | 5 | 0 | 1 |
| O146 (5) | H21, H28 | 3 | 1 | 1 | 0 | 5 | 0 | 4 | 1[d] | 0 |
| O163 (1) | H19 | 0 | 1 | 0 | 0 | 1 | 0 | 1 | 0 | 0 |
| ONT (3) | ND | 2 | 0 | 1 | 1 | 3 | 1 | 2 | 0 | 0 |
| O-rough (17) | ND | 13 | 1 | 3 | 8 | 12 | 1 | 14 | 2 | 0 |
| Total (89) | | 59 | 11 | 19 | 54 | 82 | 6 | 72 | 7 | 4 |

[a]ONT= O-antigen not typable; O-rough= rough LPS, O-antigen not typable.
[b]NM= nonmotile; ND= H-antigen not determined; NT= H-antigen not typable.
[c]HUS= hemolytic uremic syndrome.
[d]A case of double infection with *Salmonella enterica* sp.
[e]A case of a double infection with STEC O26:H11 and O157:H7.

were not identified. However, outbreak investigations were not routinely performed, and a number of these infections could be part of unidentified outbreaks. In four cases, stool specimens from persons who were in contact with the patients were examined, and two outbreaks in families with diarrheal episodes were detected. One outbreak occurred in a family living in Berlin; diarrheal symptoms developed in the parents and their two children after they returned from vacation on a farm in Northern Germany. STEC O145:H- (Stx2 and *eae*A positive STEC) could be isolated from two family members. The second outbreak was detected in a family living in the region of Augsburg (Bavaria). The parents had no diarrheal symptoms, but their children had diarrhea over a period of 6 weeks. STEC O-rough, Stx1, and *eae*A-positive STEC were isolated from stool samples of both infants. All patients from both outbreaks recovered.

### Conclusions and Recommendations

Human pathogenicity of *E. coli* strains belonging to the STEC group varied according to serotypes, virulence attributes, and other unknown factors (7). Besides Shiga toxins, the *eae*A gene product intimin contributed to diarrheal disease in humans, and typical human virulent STEC (e.g., enterohemorrhagic *E. coli* O157 strains) were found to carry genes for Shiga toxin(s), intimin, and enterohemolysin (7). Two major groups of non-O157 STEC strains were isolated from humans. Group I consisted of 54 strains (60.7%), which were all positive for *eae*A and with one exception for enterohemolysin, and group II of 35 (39.3%) STEC strains lacking the *eae*A gene. Group I STEC strains were more frequently associated with severe diarrheal disease, HUS, and with young age than group II strains, which were more frequently found in clinically uncomplicated cases (asymptomatic carriers, abdominal pain, uncomplicated diarrhea) and in older patients. These findings indicate that some or all of the group II strains are less virulent or nonpathogenic for humans, although they can colonize the human intestine. Many *eae*A-negative non-O157 STEC strains isolated from healthy animals have been found to adhere to HEp-2 cells in culture without carrying DNA sequences specific for diffuse adherence (*daa*), local adherence (*eaf*), or for enteroaggregative

Table 2. Age of patients and STEC type (*eae*A)

| Patient age (years)[a] | No. of patients with STEC-isolate | |
|---|---|---|
| | *eae*A-positive | *eae*A-negative |
| 0-1 | 6 | 1 |
| 1-2 | 13 | 1 |
| 2-3 | 11 | 0 |
| 3-4 | 5 | 2 |
| 4-5 | 2 | 1 |
| 5-6 | 4 | 0 |
| 6-10 | 2 | 2 |
| 10-15 | 2 | 4 |
| 15-20 | 0 | 4 |
| 20-30 | 2 | 2 |
| 30-40 | 3 | 5 |
| 40-50 | 4 | 2 |
| 50-60 | 0 | 6 |
| 60-72 | 0 | 1 |
| Total | 54 | 31 |

[a]Patient's age was known in 85 cases. The youngest patient was 3 months of age; the oldest, 72 years of age.

*E. coli* (14). Characterizing the colonization mechanisms of *eae*A-negative STEC could help to further define their role as human pathogens.

Our finding that infections with *eae*A-positive non-O157 STEC were more frequent in young infants resembles EPEC infection-related finding. EPEC cause gastroenteritis in very young children and occur rarely in adults. Humans exposed to EPEC show an immune response against bacterial antigens such as LPS, intimin, and fimbriae and may thus develop protective immunity to these pathogens (15). Similarly, a Canadian study has shown that non-O157 STEC-infections at young ages may confer protective immunity to subsequent infections in humans (16). It is possible that EPEC infections in early childhood confer cross-reacting protective immunity against STEC types that share common antigens (such as LPS and intimin) with classical EPEC strains. This could explain why infections with *eae*A-positive STEC occur less frequently in older patients.

Our data support previous findings that non-O157 STEC types are more frequently involved in nonbloody diarrhea than STEC-O157, whereas the latter are more frequently associated with HC and HUS (7). In 1996, 89 non-O157 STEC and 33 STEC O157 from 121 human patients were investigated in our laboratory. The proportion of patients with HUS or HC was 45.5% in the group of STEC O157 infected compared with 12.4% in the group of non-O157 STEC-infected patients. Only one of the 33 STEC O157 strains from human patients belonged to the sorbitol-fermenting, ß-glucuronidase-positive type of O157:H- strains reported in Germany (17).

Humans of all age groups can be infected with STEC belonging to different serotypes. Because the association between serotype and human pathogenicity is not always certain, a serotype-independent diagnosis of STEC is required. Although it is not possible for economic reasons to examine all patients with diarrhea for STEC, these organisms should be sought in pediatric patients, who are at higher risk for serious infection with these pathogens.

Dr. Beutin is head of the Pathogenic *E. coli* and Enterobacteria Unit at the Robert Koch-Institut. He is also a microbiologist and lecturer at the veterinary department of the Free University of Berlin. His expertise is in microbiology and molecular biology with particular interest in virulence markers of intestinal pathogenic bacteria, ecology of pathogenic bacteria, and interactions between host and pathogens.

### References

1. Calderwood SB, Acheson DWK, Keusch T, Barrett TJ, Griffin PM, Strockbine NA, et al. Proposed new nomenclature for SLT (VT) family. American Society for Microbiology News 1996;62:118-9.
2. Karmali MA. Infection by verocytotoxin-producing *Escherichia coli*. Clin Microbiol Rev 1989;2:15-38.
3. Griffin PM, Tauxe RV. The epidemiology of infections caused by *Escherichia coli* O157:H7, other enterohemorrhagic *E. coli*, and the associated hemolytic uremic syndrome. Epidemiol Rev 1991;13:60-98.
4. World Health Organization, Food Safety Unit Consultations and Workshops. Prevention and control of enterohaemorrhagic *Escherichia coli* (EHEC) infections. Report of a WHO consultation; Geneva, Switzerland; 1997 28 Apr-1 May. Geneva: The Organization; 1997. Report No.: WHO/FSF/FOS/97.6.
5. Tarr PI. *Escherichia coli* O157:H7: Clinical, diagnostic, and epidemiological aspects of human infection. Clin Infect Dis 1995;20:1-10.
6. World Health Organization. Veterinary Public Health Unit. Report on a WHO working group meeting on shiga-like toxin producing *Escherichia coli* (SLTEC) with emphasis on zoonotic aspects; Bergamo, Italy; 1994 1 Jul. Geneva: The Organization; 1995. Report No.: WHO/CDS/VPH/94.136.

7. Nataro JP, Kaper JB. Diarrheagenic *Escherichia coli*. Clin Microbiol Rev 1998;11:142-201.
8. Beutin L, Zimmermann S, Gleier K. Rapid detection and isolation of Shiga-like toxin (Verocytotoxin)-producing *Escherichia coli* by direct testing of individual colonies from washed sheep blood agar plates in the VTEC-RPLA assay. J Clin Microbiol 1996;34:2812-4.
9. Karch H, Meyer T. Single primer pair for amplifying segments of distinct shiga-like toxin genes by polymerase chain reaction. J Clin Microbiol 1989;27:2751-7.
10. Beutin L, Horbach I, Zimmermann S, Gleier K. Comparative evaluation of different diagnostic methods for the detection of verotoxin (shiga-toxin) producing strains of *Escherichia coli* (VTEC) in human clinical stool specimens. J Lab Med 1997;21:537-46.
11. Beebakhee G, Louie M, De Azavado J, Brunton J. Cloning and nucleotide sequence of the *eae* homologue from enterohemorrhagic *Escherichia coli* serotype O157:H7. FEMS Microbiol Lett 1992;91:63-8.
12. Schmidt H, Plaschke B, Franke S, Rüssmann H, Schwarzkopf A, Heesemann J, et al. Differentiation in virulence patterns of *Escherichia coli* possessing *eae* genes. Med Microbiol Immunol (Berl) 1994;183:23-31.
13. Orskov F, Orskov I. Serotyping of *Escherichia coli*. Methods in Microbiology 1984;14:43-112.
14. Beutin L, Geier D, Zimmermann S, Karch H. Virulence markers of shiga-like toxin-producing *Escherichia coli* strains originating from healthy domestic animals of different species. J Clin Microbiol 1995;33:631-5.
15. Donnenberg MS. Enteropathogenic *Escherichia coli*. In: Blaser MJ, Smith PD, Ravdin JI, Greenberg HB, Guerrant RL, editors. Infections of the gastrointestinal tract. New York: Raven Press; 1995. p. 709-26.
16. Wilson JB, Clarke RC, Renwick SA, Rahn K, Johnson RP, Karmali MA, et al. Verocytoxigenic *Escherichia coli* infection in dairy farm families. J Infect Dis 1996;174:1021-7.
17. Gunzer F, Böhm H, Rüssmann H, Bitzan M, Aleksic S, Karch H. Molecular detection of sorbitol-fermenting *Escherichia coli* O157 in patients with hemolytic-uremic syndrome. J Clin Microbiol 1992;30:1807-10.

## References

[PDF_00390] Beebakhee G., Louie M., De Azavedo J., Brunton J. (1992). Cloning and nucleotide sequence of the eae gene homologue from enterohemorrhagic Escherichia coli serotype O157:H7.. FEMS Microbiol Lett.

[PDF_00400] Beutin L., Geier D., Zimmermann S., Karch H. (1995). Virulence markers of Shiga-like toxin-producing Escherichia coli strains originating from healthy domestic animals of different species.. J Clin Microbiol.

[PDF_00330] Griffin P. M., Tauxe R. V. (1991). The epidemiology of infections caused by Escherichia coli O157:H7, other enterohemorrhagic E. coli, and the associated hemolytic uremic syndrome.. Epidemiol Rev.

[PDF_00412] Gunzer F., Böhm H., Rüssmann H., Bitzan M., Aleksic S., Karch H. (1992). Molecular detection of sorbitol-fermenting Escherichia coli O157 in patients with hemolytic-uremic syndrome.. J Clin Microbiol.

[PDF_00328] Karmali M. A. (1989). Infection by verocytotoxin-producing Escherichia coli.. Clin Microbiol Rev.

[PDF_00394] Schmidt H., Plaschke B., Franke S., Rüssmann H., Schwarzkopf A., Heesemann J., Karch H. (1994). Differentiation in virulence patterns of Escherichia coli possessing eae genes.. Med Microbiol Immunol.

[PDF_00340] Tarr P. I. (1995). Escherichia coli O157:H7: clinical, diagnostic, and epidemiological aspects of human infection.. Clin Infect Dis.

[PDF_00408] Wilson J. B., Clarke R. C., Renwick S. A., Rahn K., Johnson R. P., Karmali M. A., Lior H., Alves D., Gyles C. L., Sandhu K. S. (1996). Vero cytotoxigenic Escherichia coli infection in dairy farm families.. J Infect Dis.

